# Substance Use and HIV Risk in a Syndemic Context: Vulnerabilities Among African Refugee Sex Workers in Italy

**DOI:** 10.21203/rs.3.rs-7882022/v1

**Published:** 2025-10-21

**Authors:** Samira Shirzaei Nichols, Gamji Rabiu Abu-Ba’are, Carolina Rigo, Marco Barracchia, Al-Mardhiyyah Adams, Osman Wumpini Shamrock, Saina Abolmaali, LaRon Nelson

**Affiliations:** 1 Computer Information Systems and Analytics Department, College of Business, University of Central Arkansas, Conway, Arkansas, United States; 2 Behavioral, Sexual, and Global Health Lab, School of Nursing, University of Rochester Medical Center, Rochester, New York, United States; 3 Pink Refugees, City of Verona, Verona, Italy; 4 Department of International Health, Care and Public Health Research Institute (CAPHRI) Maastricht University, Maastricht, The Netherlands; 5 School of Nursing, Yale University, New Haven, Connecticut, United States; 6 Center for Interdisciplinary Research on AIDS, School of Public Health, Yale University, New Haven, Connecticut, United States

**Keywords:** HIV Vulnerability, Substance Use, Sex Work, Refugees and Migrants’ Health, Stigma and Discrimination

## Abstract

Substance use is a recognized driver of HIV vulnerability among sex workers, yet little is known about how behavioral patterns intersect with structural exclusion for African refugee male sex workers (RMSWs) in Europe. Guided by syndemic theory, we conducted an explanatory mixed-methods study in partnership with a community-based organization in Northern Italy. The qualitative strand included 20 in-depth interviews and two focus group discussions exploring experiences of substance use, stigma, and healthcare engagement. The quantitative strand comprised a structured survey of 150 RMSWs assessing changes in substance use before and after entering sex work and its associations with HIV-related outcomes.

Substance use increased significantly following entry into sex work, with the largest rise observed for injectable drugs. Logistic regression and chi-square analyses revealed distinct patterns by substance type: injection use was associated with lower odds of STI disclosure, reduced enjoyment of sex work, and decreased willingness to use HIV self-testing, whereas non-injection use was linked to greater HIV service awareness, higher odds of disclosure, but also increased engagement in incentivized condomless sex. Chi-square tests further indicated that escalating tobacco, alcohol, and drug use were consistently associated with higher reports of healthcare stigma, immigration-related barriers, and reduced voluntary healthcare access.

Qualitative findings contextualized these patterns, highlighting how stigma, financial precarity, and institutional exclusion shaped substance use trajectories and HIV risk. Integrated analysis underscores the need for interventions addressing structural discrimination and immigration-related barriers alongside behavioral HIV prevention for refugee sex workers.

## Introduction

Substance use is a critical yet underexplored driver of HIV vulnerability in marginalized populations. It may increase risk by contributing to condomless sex, especially during transactional or coerced encounters, impairing decision-making under the influence, and discouraging engagement with prevention and care due to stigma, criminalization, or fear of legal repercussions [2–4]. While these mechanisms are increasingly documented, little is known about how substance use specifically shapes HIV risk among African male refugee sex workers, a population that remains largely invisible in European public health research and programming.

This study is grounded in Syndemic Theory, which posits that multiple co-occurring epidemics, such as substance use, HIV, and mental health disorders, interact synergistically in populations experiencing social and structural marginalization [4]. These interactions are not merely additive but are shaped by, and contribute to, social determinants such as criminalization, stigma, poverty, and healthcare exclusion. For African refugee sex workers in Italy, who are situated at the nexus of racialized, legal, and occupational exclusion, the syndemic of substance use and HIV vulnerability is intensified by limited healthcare access, institutional stigma, and precarity tied to immigration status. This framework enables a holistic interpretation of the data, revealing how structural forces and psychosocial distress exacerbate behavioral risk, and vice versa. It underscores the need for multi-level interventions that address both behavioral health and the broader socio-political context.

Among African refugee sex workers in Italy, syndemic theory provides a lens through which to understand how substance use and HIV vulnerability are not isolated phenomena, but embedded within a nexus of systemic factors: criminalization of sex work, racialized stigma, precarious legal status, and exclusion from health services. These elements interact to amplify each other, worsening both physical and psychological outcomes.

In this population, substance use may emerge as a coping mechanism in response to trauma, discrimination, or economic vulnerability. HIV risk is simultaneously heightened by limited access to prevention tools, low health literacy, and sexual violence or coercion. Mental health distress, including depression and Post-traumatic stress disorder (PTSD), is both a contributor to and consequence of these challenges, while healthcare avoidance stems from fear of deportation, past experiences of discrimination, and the informal criminalization of sex work.

**Fig. 1:**
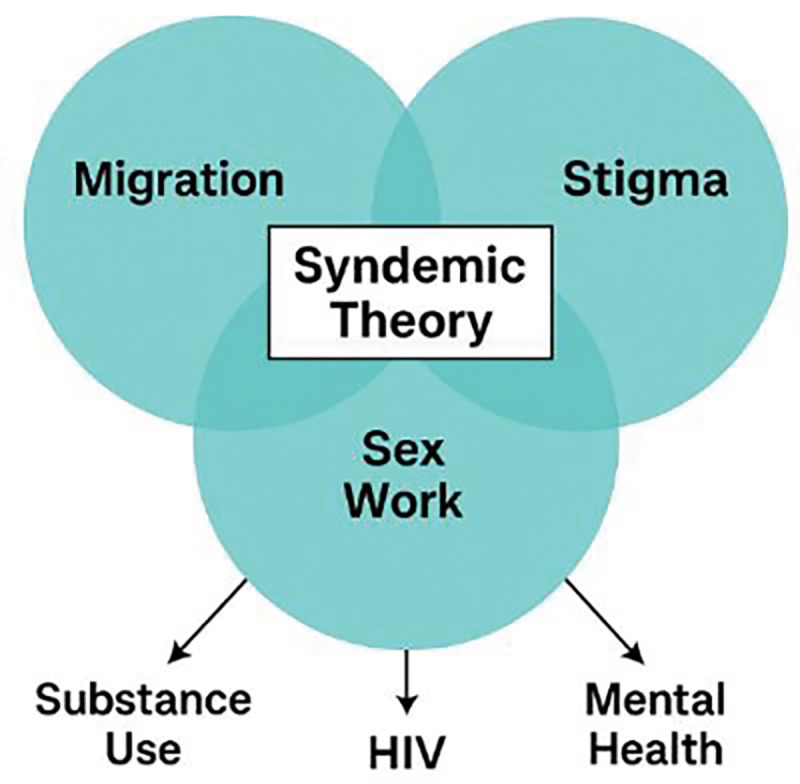
Syndemic interactions among substance use, HIV vulnerability, and structural determinants (adapted for African refugee sex workers in Italy)

Syndemic theory, therefore, shifts the analytical focus from individual behavior change to structural transformation. It advocates for multi-layered interventions that integrate harm reduction, mental health support, legal protection, and culturally appropriate services. This holistic approach is essential for breaking the cycle of co-occurring vulnerabilities and improving long-term health outcomes. The Fig. 1 provides a visual representation of how Syndemic Theory operates within this context.

This conceptualization offers a critical foundation for interpreting both quantitative and qualitative findings in this study and reinforces the need for policies that address structural determinants alongside health interventions.

Although growing recognition exists of the syndemic relationship between substance use and HIV, most research has focused on cisgender female sex workers, with comparatively little attention paid to male and gender-diverse individuals, particularly those with refugee or migrant backgrounds. Syndemic theory frames this analysis by emphasizing the intersection of substance use, HIV risk, and structural violence, including criminalization, stigma, and exclusion from healthcare, as reinforcing epidemics that disproportionately affect marginalized communities. This study expands on the syndemic framework by focusing on African refugee male sex workers, an intersectional population underrepresented in existing literature [4–9]. National data systems often fail to disaggregate by migration status, country of origin, or engagement in sex work [10]. In Italy, for example, national HIV surveillance reports provide incidence data for “foreigners” (*stranieri*), including both regular and irregular migrants, but do not disaggregate by legal status, gender identity, or type of sex work, limiting the visibility of highly marginalized subgroups and hindering the design of targeted responses [11]. Clinical evidence further shows that migrants facing structural marginalization are more likely to be lost to follow-up and to present with detectable viral loads [12].

The present study addresses this gap by focusing on African refugee sex workers in Italy, a population situated at the intersection of multiple forms of exclusion. In this context, the term “refugee” is used inclusively to refer not only to those with formal asylum but also to individuals with temporary, precarious, or undocumented legal status, whose marginal position in the Italian system compounds their exposure to violence, criminalization, and health exclusion [13]. These vulnerabilities are further intensified by racialized stigma and the informal criminalization of sex work [14]. Although selling sex is not illegal, the 1958 Merlin Law bans brothels and third-party involvement, effectively pushing sex workers into unsafe street-based or hidden indoor settings and limiting access to housing, protections, and health services [15].

Migrant sex workers, particularly those from Sub-Saharan Africa, frequently report trauma, police repression, mental distress, and substance use, often disengaging from health services even when outreach is available [13]. In several regions, local regulations further penalize street-based sex work and criminalize collective organizing, reinforcing isolation and exposure to violence [16]. While specialized services can mitigate some of these risks, their reach remains limited and uneven across the country [13].

While quantitative studies have documented associations between substance use and HIV vulnerability among sex workers, less is understood about the lived experiences of African refugee male sex workers that explain these patterns. Similarly, qualitative accounts highlight stigma, trauma, and exclusion, yet few studies integrate these perspectives with quantitative trends. To date, the two strands have rarely been combined to provide a fuller understanding of how substance use trajectories intersect with HIV risk in refugee contexts. This study examines the relationship between substance use and HIV vulnerability among African refugee sex workers in Italy. Specifically, it investigates how patterns of drug and alcohol use change before and after entry into sex work, and explores associations between substance use, access to care, health behaviors, and psychosocial outcomes.

Using an exploratory mixed-methods approach, this study combines quantitative survey data with qualitative interviews to capture both behavioral patterns and the broader structural context shaping HIV risk. Specifically, it aimed to:

1. **Qualitative strand:** Explore how African refugee sex workers describe their substance use, stigma, and healthcare experiences in Italy.
2. **Quantitative strand:** Assess changes in substance use before and after entering sex work and examine associations with HIV-related behaviors, healthcare access, and psychosocial outcomes.
3. **Integration question:** Determine how qualitative narratives explain or challenge quantitative trends in substance use and HIV vulnerability.

This design provides a comprehensive understanding of how substance use intersects with migration, sex work, and healthcare access within a marginalized population. The findings are intended to inform evidence-based, community-driven interventions grounded in the lived experiences of African refugee sex workers in Italy. To our knowledge, this is the first mixed-methods analysis of African refugee male sex workers in Europe.

## Background

Substance use plays an important role in increasing HIV vulnerability among African refugee sex workers in Italy. This group faces compounded risks stemming from their migration status, involvement in sex work, and limited access to healthcare and social support systems. The literature consistently shows that HIV vulnerability in this population is shaped by a complex interplay of individual behavior and structural forces.

### Substance Use and HIV Risk Among Refugee Sex Workers

The correlation between drug use and HIV transmission is well-established across regions. Talbott found that the number of HIV-infected sex workers in a country significantly predicts national HIV prevalence levels, reinforcing the need to target this population for HIV prevention [17]. Tuan et al. reported a 16.3% HIV prevalence among street-based sex workers in Ho Chi Minh City, with injecting drug use and young age being the key predictors, paralleling dynamics seen among marginalized refugee populations in Italy [18].

Roxburgh et al. documented how female street-based sex workers in Sydney experienced high levels of drug dependence and physical violence, along with limited condom use, particularly with non-paying partners [19]. Similar findings were reported by Inciardi et al. in Miami, where drug-involved sex workers continued to engage in risky sex despite knowing their HIV status, underscoring how addiction limits behavioral change [20]. Paone et al. also observed higher rates of injection drug use and risky sexual behavior among current sex workers in methadone maintenance programs, indicating that drug use is a persistent barrier to safe practices [21].

In the Caribbean Basin, Surratt found that substance use and HIV risk among migrant female sex workers were intensified by displacement, unstable housing, and restricted healthcare access [22]. These findings align with the Italian context, where undocumented African sex workers are driven to survival sex under exploitative conditions. Rusakova et al. emphasized that substance-using sex workers are at a markedly increased risk of HIV due to a convergence of behavioral, social, and structural factors [23]. The study highlighted how drug and alcohol dependence impair decision-making and reduce condom negotiation power, particularly under withdrawal pressure or coercion by pimps or clients. Injection drug use introduces a dual transmission risk: unprotected sex and needle sharing, making this subgroup exceptionally vulnerable. This insight reinforces the urgent need for structural and harm reduction responses, especially in refugee contexts where agency is already constrained.

### Drug Use, Risky Sexual Behavior, and Structural Barriers

A significant body of research underscores how drug use shapes daily routines and sexual practices among sex workers. In South Africa, studies by Parry et al. and Needle et al. documented the use of substances such as heroin, cocaine, and Mandrax to cope with the demands of sex work, attract clients, and dissociate from trauma [24–25]. These substances are often exchanged for sex or used under pressure, undermining sex workers’ ability to negotiate condom use, especially in contexts marked by violence and power imbalances.

In the Netherlands, van Veen et al. found that HIV prevalence reached 13.6% among drug-using female sex workers and 18.8% among transgender sex workers, compared to just 1.5% among non-drug-using women [26]. Importantly, 74% of HIV-positive participants were unaware of their status, pointing to critical gaps in access to prevention services. Although sex work is legal in the Netherlands, the findings reveal that legal reforms alone are insufficient to protect the most marginalized. As Dekker, Tap, and Homburg explain, the legalization of prostitution has not translated into improved social status or healthcare access for sex workers [27]. These patterns are also evident in Italy. Marrone et al. studied 936 African refugees in Lazio and found a high burden of neglected tropical diseases such as schistosomiasis (31%) and hepatitis B (10.8%), alongside a lower but still relevant HIV prevalence of 0.7% [28]. Additionally, Barracchia et.al emphasize that the lack of coherent national harm reduction policies in Italy contributes to heightened HIV vulnerability, especially among individuals at the intersection of multiple stigmatized identities, such as refugees and sex workers [29]. While refugees often arrive in good health, many develop chronic conditions due to systemic neglect, diagnostic gaps, and poor follow-up care, conditions that equally affect HIV detection and treatment.

### Complex Power Dynamics and Gendered Risk

Power dynamics between sex workers and clients, pimps, or intimate partners often reinforce substance dependency and HIV risk. Parry et al. and Needle et al. both highlighted how many sex workers are controlled by abusive partners or pimps who are also their drug suppliers, effectively stripping them of autonomy [24–25]. These relationships often involve forced unprotected sex, physical violence, and economic coercion.

Yu and Li’s systematic review of 41 studies reinforced that HIV risk in drug-using sex workers is rarely a matter of isolated behavior. Instead, it stems from overlapping vulnerabilities such as criminalization, poverty, and stigma [30]. Gossop et al. similarly found that among female drug users in London, those engaged in sex work showed markedly different risk profiles than non-sex-working peers, with higher rates of heroin and cocaine use and lower rates of protective behaviors [31].

These gendered dynamics are compounded for African refugee sex workers in Italy, who often operate under irregular immigration status and face racialized stigma. Their lack of access to public health infrastructure and legal protection leaves them particularly vulnerable to both sexual exploitation and HIV transmission.

### Evidence from Intervention and Prevention Studies

Despite these vulnerabilities, some interventions show promise. Carney et al. evaluated the Ithubalethu project in South Africa, a harm reduction intervention aimed at drug-using sex workers. The program led to reduced frequency of drug use and increased awareness of HIV risks, demonstrating the effectiveness of culturally grounded, community-based approaches [25], [32].

However, such interventions remain rare, particularly in European contexts dealing with migrant populations. Surratt’s work in the Caribbean Basin emphasized the need for tailored programming that accounts for the dual stigmas of sex work and migration [22]. Paone et al. and Inciardi et al. also called for integrated strategies that address both addiction and sexual health simultaneously [20–21].

## Conclusion

Substance-using sex workers, particularly those facing socio-legal exclusion, are at substantially higher risk of HIV. Structural factors such as poverty, stigma, gendered violence, and criminalization, rather than individual behaviors alone, drive this risk. As Rusakova et al. note, behavioral risk is magnified when layered with institutional disempowerment, withdrawal distress, and forced unprotected sex [23]. For African refugee sex workers in Italy, the combination of racialized discrimination, immigration status, and substance dependency magnifies these dangers. Effective interventions must address both behavioral and structural dimensions through decriminalization, inclusive healthcare, and harm reduction strategies grounded in the lived experiences of this population. While quantitative evidence underscores the high prevalence of substance use and HIV risk among sex workers, and qualitative studies reveal structural barriers and coping mechanisms, these strands have rarely been integrated. In particular, little is known about how changes in substance use before and after entering sex work interact with structural exclusion to shape HIV risk for African refugee sex workers in Europe.

## Methods

### Overall Study Design

An exploratory mixed-methods design was employed [33]. Qualitative findings guided the development of the quantitative survey, while integration occurred during the interpretation stage to refine and contextualize statistical trends. This design was chosen to compare patterns of substance use and HIV vulnerability with lived experiences of stigma and healthcare engagement, thereby enhancing both the breadth and depth of understanding. Guided by syndemic theory, the mixed-methods approach captured not only the behavioral dimensions of substance use but also their intersections with psychosocial and structural determinants. The qualitative strand complemented the quantitative analysis, offering deeper insight into stigma, trauma, and institutional exclusion.

### Participants and Setting

The study was conducted in partnership with Circolo Pink (Pink Refugees), a community-based nonprofit organization with established trust and access to refugee and migrant sex worker communities in Italy. Data collection took place in safe and accessible spaces at their main office in Verona and at a sister institution in Turin, Northern Italy. Eligibility criteria required participants to:

1. Be refugees from Sub-Saharan Africa
2. Currently reside in Italy
3. Be 18 years of age or older
4. Demonstrate fluency in English or Italian
5. Self-identify as sex workers
6. Have engaged in sex work within the past six months

### Data Collection Procedures

#### Qualitative Strand.

We conducted **20 in-depth interviews (IDIs)** and **two focus group discussions (FGDs)** with African refugee and migrant sex workers (RMSWs). These sessions explored lived experiences, barriers, and facilitators related to substance use and HIV vulnerability.

#### Quantitative Strand.

Findings from the qualitative strand informed the development of a **structured survey**, which was administered to **150 RMSWs** to assess the generalizability of qualitative insights, particularly regarding changes in substance use before and after entry into sex work. Surveys were implemented using **REDCap**, a secure mobile platform.

#### Recruitment.

Given the hidden nature of the population, recruitment relied on **venue-based and snowball sampling** through Pink Refugees. Two trained **peer-research assistants**, themselves RMSWs, facilitated recruitment by engaging peers during weekly community meetings. Additional participants were recruited through referrals from FGD participants and survey respondents.

#### Data Management.

All IDIs and FGDs were conducted in person at Pink Refugees and its sister site, audio-recorded with participant consent, and supplemented with detailed field notes.

### Qualitative Data Analysis

Interview and focus group transcripts were reviewed to identify recurring themes related to substance use, stigma, and HIV vulnerability. A thematic approach was applied, guided by the study’s key domains:

1. Substance use in the context of sex work
2. Coping with stigma and emotional challenges
3. Experiences with chemsex and hard drug use
4. Barriers to HIV testing and health services

Themes were compared across participants to highlight both shared experiences and divergent perspectives.

### Qualitative Data Analysis

Analysis followed a summative content analysis approach [34], incorporating interpretive thematic synthesis as described by Braun and Clarke [35]. All in-depth interviews (IDIs) and focus group discussions (FGDs) were audio-recorded with participant consent, transcribed verbatim by trained peer research assistants, and deidentified to ensure confidentiality. When interviews were conducted in Italian or English, bilingual team members cross-checked translated segments to preserve cultural and semantic accuracy. We employed a summative content analysis approach, which combines counting the frequency of key words or phrases with interpretive analysis of their contextual meaning. This method was chosen to identify recurring expressions related to HIV testing, stigma, and healthcare access while situating them within broader socio-structural experiences. Two trained analysts reviewed each transcript independently, highlighting recurring terms, ideas, and expressions that reflected barriers, facilitators, or lived experiences of HIV testing and care. These patterns were tabulated using Excel to record word frequencies and contextual notes. Coding, in this approach, functioned as an *intermediate step* to group related expressions rather than as an exhaustive codebook process. Analysts developed short analytic summaries (approximately 150–200 words per transcript) synthesizing recurrent ideas and illustrative quotations. The lead analyst compiled these into a summary matrix that organized key themes by frequency and context. Through iterative team discussions, the matrix was refined to ensure conceptual clarity and consistent interpretation across transcripts. This process maintained the analytic rigor of qualitative inquiry while allowing quantification of pattern strength across participants.

### Trustworthiness and Reflexivity

To enhance trustworthiness, the team engaged in ongoing peer review and triangulation across IDIs and FGDs. The analytic process was documented through memo writing and discussion logs, creating an audit trail of analytic decisions. Summaries were shared with Circolo Pink staff for interpretive feedback and contextual validation, ensuring community perspectives informed final interpretations.

The research team’s reflexivity was central throughout the analysis. The qualitative analysts included two peer research assistants, both African refugee sex workers affiliated with Circolo Pink, whose lived experience informed the interpretation of stigma, discrimination, and healthcare exclusion. The principal investigator, an external researcher, maintained a reflexive stance through analytic journaling and regular consultation with community partners to mitigate outsider bias.

This summative approach allowed the team to identify how frequently particular experiences were described while also interpreting their deeper social meanings, linking participants’ narratives to the syndemic framework guiding the study.

### Quantitative Data Analysis

All quantitative analyses were conducted using **RStudio (version 2025.05.1)**. Analyses proceeded in three stages: descriptive, inferential, and multivariate modeling.

#### 1. Descriptive Statistics

We first summarized key demographic characteristics (e.g., age, gender, nationality) and behavioral variables (e.g., types of substances used) to describe the study population. Substances were categorized as either **injectable** or **non-injectable**. When the route of administration was unclear, substances were conservatively classified as injectable to account for potential risk implications.

#### 2. Repeated-Measures ANOVA

To assess changes in substance use before and after entry into sex work, we conducted a repeated-measures analysis of variance (ANOVA) using the *ezANOVA()* function from the *ez* package in R (version 4.4.3). The within-subject factors were:

- **Time:** before vs. after entering sex work
- **Substance type:** injectable vs. non-injectable

The dependent variable was the number of substances used. Effect sizes were reported as generalized eta squared (ges) for each main effect and the interaction, providing estimates of variance explained.

#### 3. Multivariate analyses of covariance (MANCOVA)

For multivariate models, we used Pillai’s trace as a robust test statistic, as it performs well under unequal covariance assumptions. Partial eta squared (*η*^2^) was calculated as an effect size measure with 95% confidence intervals. In models including multiple correlated predictors and outcome domains, large values of Pillai’s trace may align numerically with *η*^2^, reflecting the shared explained variance across dependent variables rather than redundancy in metrics.

#### 4. Logistic and Multinomial Logistic Regression

To examine associations between post–sex work substance use and health-related, behavioral, and psychosocial outcomes, we employed logistic regression models. Binary logistic regression was applied to assess whether injection or non-injection drug use predicted outcomes such as healthcare access, STI-related behaviors, service satisfaction, and sexual health practices. When dependent variables had more than two unordered categories, multinomial logistic regression was used.

#### 5. Chi-Square Tests of Independence

To further explore associations between substance use patterns and HIV vulnerability, chi-square tests of independence were conducted. These tests assessed relationships between categorical variables, including condom use, STI history, access to healthcare, and experiences of discrimination. When necessary, Fisher’s exact test was applied to account for sparse cell counts. Bivariate findings from these tests informed subsequent multivariate modeling.

### Integration of Quantitative and Qualitative Data

Integration occurred during the interpretation phase through side-by-side comparison and the construction of joint displays aligning quantitative associations (e.g., HIV testing frequency, disclosure, healthcare access) with qualitative themes (e.g., stigma, institutional exclusion, coping with substance use). The integration process followed an explanatory sequential logic: qualitative findings from interviews and focus group discussions were first used to inform the development of the quantitative survey, while the final stage of analysis involved merging and comparing results across strands. Side-by-side narratives were used to highlight areas of convergence (e.g., both strands linking substance use to reduced voluntary healthcare engagement), complementarity (e.g., qualitative narratives explaining mechanisms behind quantitative trends), and divergence (e.g., discrepancies between observed HIV testing rates and lived experiences of compulsory testing). The qualitative strand contributed contextual depth, revealing how stigma, immigration barriers, and trauma shaped substance use trajectories and HIV vulnerability, while the quantitative strand provided statistical evidence of associations between substance use, health behaviors, and service access. Integrating the two strands enhanced explanatory power and offered a more comprehensive understanding of the syndemic interaction between behavioral and structural factors influencing HIV risk among African refugee sex workers in Italy. Reporting followed the Good Reporting of a Mixed Methods Study (GRAMMS) framework to ensure transparency in integration and interpretation [36].

## Results

This section presents the study’s findings in three parts, beginning with qualitative insights and followed by quantitative analyses and integrated results. The qualitative results explore how African refugee sex workers experience and interpret substance use, HIV vulnerability, and structural barriers within the broader context of forced migration and stigma. These narratives provide a foundational understanding of the complex interplay between behavioral and social forces. The subsequent quantitative results build on these themes, statistically examining associations between substance use patterns, healthcare access, stigma experiences, and HIV-related risk factors. Together, these findings offer an integrated perspective on the syndemic factors shaping HIV vulnerability in this marginalized population.

### Qualitative Insights: Substance Use and HIV Vulnerability

#### Substance Use in the Context of Sex Work

The majority of participants reported abstaining from drug or alcohol use during sexual encounters. However, substance use, particularly by clients, was a recurrent concern, especially in the context of sex parties. Several participants described feeling unsafe or uncomfortable in these situations, emphasizing the perceived risks associated with clients under the influence. For example, one participant reflected on past experiences, recounting “dangerous encounters with clients under the influence of drugs.”

Although most participants personally avoided substances during work, a few disclosed knowing peers who turned to drug use as a coping mechanism. One interviewee noted, “I meet with a Nigerian guy who got into drugs because of stigma from his friends… he said no, that’s the only thing that helps him.”

#### Coping with Stigma and Emotional Challenges

Substance use emerged as a means of dealing with the psychological toll of sex work and societal rejection. Several respondents highlighted how peers used alcohol or harder substances to escape feelings of shame or alienation. One participant shared: “Sometimes my friends drink alcohol and other hard drugs, but for me, I don’t care about what anyone says.” Another stated feeling deep regret after drinking: “I sometimes get bad when I have sex under the influence of alcohol, and when I wake up, and see the person is not up to standard. I feel bad about it, very, really bad.”

This pattern of using substances to manage stigma and internalized judgment highlights a broader emotional burden among African male sex workers, which can compound vulnerability to HIV.

#### Experiences with Chemsex and Hard Drug Use

While chemsex was not universally reported, service providers and community advocates described it as an emerging issue. One respondent from a community organization recalled “a defining moment” when a long-standing member experienced a breakdown due to hard drug use tied to sex work. Similarly, another professional acknowledged that the invisibility of chemsex users in services may reflect structural gaps in outreach and accessibility: “There are difficulties in intercepting… the population of sex workers, or people who actually use substances… [who] do not access because there is perhaps no service ready to receive them.”

This suggests a need for services to evolve in how they identify and respond to substance use patterns among marginalized populations.

#### Barriers to HIV Testing and Health Services

Fear and misinformation about HIV were dominant themes deterring participants from testing. As one participant explained, “They don’t have training regarding this disease… for them this disease leads to death.” Substance use was also implicated as a factor leading to disengagement from preventive health behaviors. “Some under the effect of substances… don’t give a damn, so for them it’s life, only one, they live as they want.”

Furthermore, access to HIV testing and care was mediated by institutional settings. Some participants were tested only upon incarceration, unaware of their HIV status prior. “When they enter the prison… they find out randomly they already have HIV but didn’t know.”

Providers also noted a lack of targeted HIV awareness campaigns. Most initiatives were described as “horizontal campaigns,” not tailored to the unique risks and cultural contexts of African male sex workers. This lack of specificity, combined with administrative barriers, such as a lack of documentation, further limits access to HIV prevention and care services.

### Quantitative Results

#### Participant Characteristics

The survey included 150 participants, primarily African male refugee sex workers in Italy. Table 1 presents a summary of their demographic characteristics. The majority of participants (96.97%) identified as male, with a median age of 30 years. Nearly half (47.33%) identified as Christian, and 57.33% reported being single. Additionally, 87.33% were refugees, and 57.33% were of Nigerian origin.

#### Classification of Injectable and Non-Injectable Substances

To assess differences in substance use risk profiles, all reported substances were categorized as *injectable* or *non-injectable*. Substances were classified as *injectable* when they are known to be commonly or potentially administered via injection in European or international epidemiologic contexts, including heroin, cocaine, methamphetamine, opioids, and certain stimulants. This approach aligns with surveillance and policy definitions used by the European Monitoring Centre for Drugs and Drug Addiction (EMCDDA), which defines *problem drug use* as injecting or long-term/regular use of opioids, cocaine, or amphetamines, and reports increasing injection of stimulants and synthetic cathinones across Europe [33–34]. The World Health Organization’s *Rapid Assessment and Response Guide on Injecting Drug Use* similarly notes that surveys often approximate route of administration when direct self-report data are unavailable, given the strong behavioral and health differences between injection and non-injection use [39]. We acknowledge that this conservative classification, assigning substances as *injectable* when injection was possible but not confirmed, may overestimate injection prevalence. However, given evidence that multiple substances are injected in European contexts, including Italy, this rule prevents underestimation of HIV-related risk exposures in this population. The list of these categories is as follows.

### Injectable Drugs

These substances are commonly or potentially administered via injection, although they may also be used in other ways (e.g., smoked or snorted):

- Heroin
- Cocaine (can be injected, but also snorted or smoked)
- Crystal Meth (can be injected, smoked, or snorted)
- Speed (amphetamines – sometimes injected)
- Pain killers when not sick (e.g., opioids like morphine or oxycodone – sometimes injected)
- MDMA (ecstasy) (rarely injected, more commonly taken orally, but possible)
- PCP (phencyclidine) (in rare cases)

### Non-Injectable Drugs

These substances are primarily used through oral, inhalation, or other non-injection methods:

- Acid (LSD – typically taken orally via blotter paper)
- Alcohol (ingested)
- Cough mixture when not sick (ingested)
- Crack (smoked)
- Edibles (marijuana-infused candies) (ingested)
- Marijuana (smoked or ingested)
- Sniffing Glue (inhaled)
- Tobacco (cigarettes, E-cigarettes, Hookah/Shisha) (inhaled/smoked)
- Alkyl nitrates (poppers) (inhaled)

#### Sensitivity Analysis for Classification Robustness

To test the robustness of our classification rule, a sensitivity analysis reclassified ambiguous substances (e.g., cocaine, methamphetamine, MDMA) as *non-injectable* based on their most common routes of administration in the European setting [38]. All primary models, repeated-measures ANOVA, logistic, and multinomial logistic regressions, were re-estimated under this alternative classification. The direction, magnitude, and significance of key associations did not change materially, indicating that the findings were robust to differences in classification criteria.

#### Repeated-Measures ANOVA

A repeated measures ANOVA was conducted to examine changes in substance use over time (before vs. after entering sex work) and by substance type (injectable vs. non-injectable). There was a significant main effect of time, *F(1, 149)* = *59.99*, *p <0.001*, generalized eta squared (*ges)* = *0.196*, indicating an overall change in substance use over time, as shown in Table 2. A significant main effect of substance type was also observed, *F(1, 149)* = *397.01*, *p* < *0.001*, *ges* = *0.061*, with participants reporting higher use of non-injectable substances (*Mean* = *5.77*, *SD* = *3.37*) compared to injectable substances (*Mean = 4.30, SD = 2.76*), averaged across time points.

**Fig. 2:**
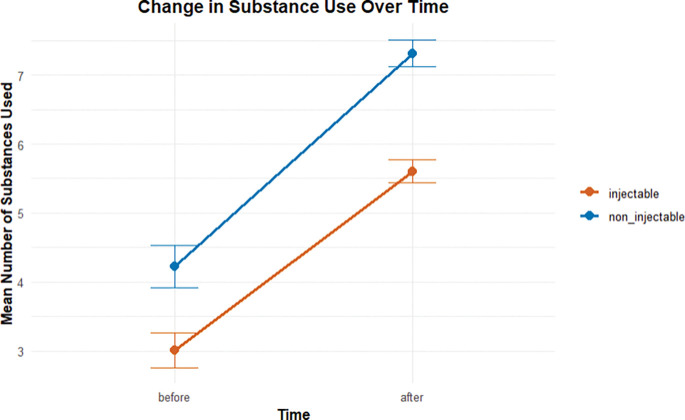
Changes in substance use before and after entering the sex-work industry

Importantly, as shown in Table 2, there was a significant time and substance use interaction, *F(1, 149) = 12.11*, *p < 0.001*, *ges = 0.002*, indicating that the pattern of change over time differed by substance type. Injectable substance use increased from before (*Mean*= *3.01*, *SD* = *3.10*) to after (*Mean* = *5.60*, *SD* = *2.04*), while non-injectable use also increased but to a lesser degree, from before (*Mean* = *4.23*, *SD* = *3.74*) to after (*Mean* = *7.31*, *SD* = *2.36*). This differential increase is illustrated in Fig. 2. (Eta squared: small: 0.01, medium: 0.06, high: 0.14)

#### Paired t-Tests

To further examine changes by substance type, paired *t*-tests were conducted separately for injectable and non-injectable substances, and the results are shown in Table 3.

- **Injectable substances**: Use after entry into sex work was significantly higher than before (*t* = 8.54, *p* < 0.001, 95% CI [1.99, 3.18]), with a mean increase from 3.01 to 5.60.
- **Non-injectable substances**: A similar pattern was observed, with significantly greater use after entry (*t* = 8.55, *p* < 0.001, 95% CI [2.38, 3.80]), increasing from 4.23 to 7.31.

Both categories of substances showed significant increases after participants entered sex work, reinforcing the repeated-measures ANOVA results and highlighting the overall escalation in substance use over time.

#### Multivariate Analysis of Covariance (MANCOVA)

A multivariate analysis of covariance (MANCOVA) was conducted to examine whether post–sex work substance use patterns (injectable and non-injectable) were associated with a combined set of behavioral, psychosocial, and healthcare-related outcomes.

Results indicated a significant multivariate effect of **injectable substance use**, *Pillai’s Trace* = 0.57, *F*(33, 101) = 4.05, *p* < .001, *partial η*^2^ = 0.57, 95% CI [0.37, 1.00], representing a large effect size.

**Non-injectable substance use** did not yield a significant multivariate effect, *Pillai’s Trace* = 0.29, *F*(33, 101) = 1.26, *p* = .192, *partial η*^2^ = 0.29, 95% CI [0.00, 1.00]. The upper confidence limit was truncated at 1.00, reflecting the bounded nature of *η*^2^.

The similarity between Pillai’s Trace and partial *η*^2^ for the injectable variable reflects the dominance of this predictor in explaining shared variance across the highly correlated dependent variables, which is expected in multivariate models with overlapping outcome domains.

#### Logistic and Multinomial Logistic Regression Results

The Logistic and multinomial logistic regression analyses were conducted to assess associations between post–sex work substance use (injectable vs. non-injectable) and a range of psychosocial, behavioral, and healthcare-related outcomes. Odds ratios (ORs) and *p*-values are reported in Table 4.

### Healthcare Access and Awareness

- Non-injection users were significantly more likely to report challenges with distance from healthcare (OR = 1.482, *p* = 0.015).
- They were also more likely to know where to go for HIV testing and care (OR = 1.384, *p* = 0.036).

### STI-Related Behaviors

- Injection users had lower odds of notifying clients if they contracted an STI (OR = 0.575, *p* = 0.006).
- In contrast, non-injection users had higher odds of disclosure (OR = 1.554, *p* = 0.013).

### Occupational and Healthcare Satisfaction

- Injection users were less likely to report enjoyment of sex work (OR = 0.483, *p* = 0.005).
- Non-injection users were more likely to report enjoyment (OR = 1.582, *p* = 0.044).
- Non-injection use was also associated with higher satisfaction with healthcare services in Italy (OR = 1.709, *p* = 0.034).

### Health Outcomes

- Non-injection use was associated with higher odds of having been diagnosed with an STI (OR = 1.559, *p* = 0.026).
- Injection users were less likely to report unmet healthcare needs (OR = 0.499, *p* = 0.008).

### Sexual Risk Behaviors

- Non-injection users had higher odds of engaging in incentivized unprotected sex (OR = 1.616, *p* = 0.005).
- Injection users had lower odds of such behavior (OR = 0.646, *p* = 0.024).

### Engagement with HIV Prevention

- Injection users were less likely to express willingness to use an HIV self-testing kit in the future (OR = 0.479, *p* = 0.020).

The findings in Table 4 reveal distinct behavioral and health-related patterns between injection and non-injection users. Non-injection drug use was generally associated with greater healthcare awareness and positive perceptions of services but also linked to higher engagement in incentivized risk behaviors. In contrast, injection drug use was associated with reduced enjoyment of sex work, lower engagement in preventive practices, and decreased willingness to adopt HIV self-testing.

#### Chi-square Independence tests

Chi-square tests of independence (and Fisher’s exact tests where assumptions were violated) were used to examine associations between increased substance use after entry into sex work and HIV-related outcomes. For statistically significant associations, **Cramér’s V** was reported as a measure of effect size (0.10 = small, 0.30 = medium, 0.50 = large).

##### 1. Substance Use and HIV Testing Frequency

Significant associations were found between increased substance use and frequency of HIV testing (Table 5).

- **Tobacco use**: *χ*^2^ = 20.39, *p* < 0.001, Cramér’s V = 0.37 (moderate).
- **Illicit drug use**: *χ*^2^ = 17.37, *p* < 0.001, Cramér’s V = 0.34 (moderate).
- **Alcohol use**: *χ*^2^ = 14.35, *p* = 0.002, Cramér’s V = 0.31 (moderate).

As we illustrated in Table 5 Participants who reported increasing substance use were more likely to test **once a year** and less likely to test quarterly or less than once a year.

##### 2. Substance Use and Healthcare Service Utilization

Significant associations emerged for alcohol and illicit drug use (Table 6).

- **Alcohol use**: *χ*^2^ = 9.19, *p* = 0.002, Cramér’s V = 0.25 (small–moderate).
- **Illicit drug use**: *χ*^2^ = 6.61, *p* = 0.010, Cramér’s V = 0.21 (small–moderate).

Participants who increased alcohol or illicit drug use were disproportionately likely to access healthcare **never or less than once a year**. The results are shown in Table 6.

##### 3. Substance Use and Stigma in Healthcare Settings

Increased use of all three categories (tobacco, alcohol, and illicit drugs) was significantly associated with higher reports of stigma or discrimination in hospitals (Table 7).

- **Tobacco use**: *χ*^2^ = 15.65, *p* < 0.001, Cramér’s V = 0.32 (moderate).
- **Alcohol use**: *χ*^2^ = 25.89, *p* < 0.001, Cramér’s V = 0.42 (large).
- **Illicit drug use**: *χ*^2^ = 9.33, *p* = 0.002, Cramér’s V = 0.25 (small–moderate).

##### 4. Substance Use and Immigration-Related Barriers to Healthcare

Substance use escalation was also associated with immigration-related difficulties when seeking healthcare (Table 8).

- **Tobacco use**: *χ*^2^ = 17.82, *p* < 0.001, Cramér’s V = 0.34 (moderate).
- **Alcohol use**: *χ*^2^ = 15.11, *p* < 0.001, Cramér’s V = 0.32 (moderate).
- **Illicit drug use**: *χ*^2^ = 9.28, *p* = 0.002, Cramér’s V = 0.25 (small–moderate).

Chi-square analyses revealed that increased use of tobacco, alcohol, and illicit drugs after entry into sex work was consistently associated with:

1. Higher likelihood of annual HIV testing (rather than more frequent testing).
2. Lower frequency of healthcare utilization.
3. Greater reports of stigma and discrimination in hospital settings.
4. More immigration-related barriers to accessing healthcare.

These findings suggest that while escalating substance use may coincide with institutionalized HIV testing, it is also linked to reduced voluntary healthcare engagement and heightened experiences of stigma and exclusion.

While the quantitative results revealed significant associations between substance use, healthcare engagement, and sexual risk behaviors, the qualitative strand provided insight into the lived experiences underlying these patterns. To integrate these perspectives, we developed joint displays aligning statistical associations with participant narratives, highlighting areas of convergence, complementarity, and divergence

### Integrated Findings

The integrated analysis revealed how substance use trajectories, when examined alongside lived experiences of stigma and healthcare engagement, provide a more comprehensive understanding of HIV vulnerability among African refugee sex workers. Quantitative analyses demonstrated significant associations between escalating substance use and HIV testing, reduced healthcare utilization, increased stigma, and immigration-related barriers, while qualitative narratives contextualized these patterns by highlighting institutional discrimination, financial precarity, and coping strategies. Together, the joint displays show that non-injection users often had higher service awareness and disclosure rates but also engaged more in incentivized risk behaviors, while injection users were more likely to experience diminished enjoyment in sex work, reduced disclosure, and disengagement from prevention technologies. These integrated findings underscore that behavioral and health outcomes cannot be understood apart from the broader structural and psychosocial contexts shaping the lives of refugee sex workers.

#### Integrated Findings on Healthcare Access and Stigma

Quantitative analyses showed that escalating substance use was significantly associated with reduced healthcare utilization, greater reports of stigma in medical settings, and immigration-related barriers to access. The qualitative strand contextualized these findings by illustrating how participants often disengaged from care under the influence of substances, described the absence of services prepared to meet their needs, and emphasized that HIV testing was frequently encountered only in institutional or compulsory settings such as prisons. Together, these results demonstrate convergence between strands on reduced engagement, complementarity in explaining systemic exclusion, and divergence in how testing was experienced, highlighting the role of structural barriers in shaping HIV vulnerability. Table 9 illustrates how these strands converge and complement one another, highlighting the compounded vulnerabilities that shape HIV risk among African refugee sex workers.

### Divergent Findings

Although quantitative analyses showed higher rates of HIV testing among participants reporting increased substance use, qualitative narratives clarified that this pattern primarily reflected **compulsory or institutional testing** rather than voluntary health-seeking behavior. Several participants recounted learning of their HIV status only after incarceration or during mandatory screening: *“When they enter the prison… they find out randomly they already have HIV but didn’t know.”*

This divergence underscores that apparent increases in testing frequency may not indicate improved access or motivation for preventive care. Instead, they reflect **structural coercion within institutional settings**, reinforcing how structural exclusion and limited healthcare autonomy shape HIV vulnerability among African refugee sex workers.

#### Integrated Findings on Sexual Risk and Disclosure Practices

Logistic regression results revealed distinct behavioral patterns by substance use type: injection users were less likely to disclose STIs, report enjoyment of sex work, or adopt HIV prevention tools, whereas non-injection users had higher odds of disclosure but were also more likely to engage in incentivized condomless sex. The qualitative findings deepened these patterns, with participants describing negative experiences of sex under the influence, substance use as a coping response to stigma, and dangerous encounters with clients in drug-related contexts. These integrated findings show convergence on the ways substance use reduces agency and increases risk, while also highlighting complementarity in explaining why non-injection users may remain more socially connected despite engaging in higher-risk practices. The Table 10 summarizes these integrations.

Taken together, the integrated analysis revealed that substance use trajectories were deeply intertwined with HIV-related health outcomes through pathways shaped by stigma, immigration barriers, and economic precarity. Quantitative results established significant associations between substance use, healthcare access, sexual risk behaviors, and disclosure practices, while qualitative narratives illuminated the mechanisms underlying these associations, from discrimination in healthcare settings and service gaps for migrants, to the role of substances in coping with stigma and managing client interactions. Importantly, integration highlighted both convergence (e.g., reduced healthcare engagement and diminished enjoyment of sex work) and divergence (e.g., apparent increases in HIV testing that reflected compulsory rather than voluntary screening). These findings underscore that behavioral risks cannot be understood apart from the structural and psychosocial conditions that frame the lives of refugee sex workers, reinforcing the value of a mixed-methods approach in capturing both patterns and lived experiences of HIV vulnerability.

## Discussion

This study identified a significant increase in both injectable and non-injectable substance use following entry into sex work among African refugee sex workers in Italy. To our knowledge, this is one of the first mixed-methods studies to quantify and contextualize differential health outcomes by substance administration route among African refugee sex workers in Italy and Europe. Our findings show that injectable drug use increased from a mean of 3.01 to 5.60, and non-injectable use from 4.23 to 7.31 (*p* < 0.001), signaling heightened HIV vulnerability due to injection-related risks and substance-influenced decision-making. This significant increase, particularly in injectable drug use (p < 0.001), supports the notion that entering into sex work is a major turning point in substance use trajectories. These trends highlight a critical moment for early intervention, particularly around initiation into sex work. From our findings, we can additionally assume that the drug administration route correlates with different health behaviors. Injectable use may indicate more severe substance dependence and social disconnection, requiring differentiated interventions. However, although one-third of sex workers in a recent review reported lifetime use of illicit drugs [40], there remains a lack of targeted guidance on service delivery for individuals who engage in both sex work and drug use, and even more specifically, on specific routes between injection and non-injection drug users [41]. It remains crucial to approach both injection and non-injection drug use for African male sex workers using multi-component and comprehensive interventions, integrating structural strategies, harm reduction, safer sex initiatives, and access to health services [42].

The findings reveal divergent patterns between injection and non-injection users. Injection drug use was linked to lower STI disclosure (OR = 0.575), reduced willingness to use HIV self-testing kits (OR = 0.479), and lower enjoyment of sex work (OR = 0.483). In contrast, non-injection users were more likely to disclose STIs (OR = 1.554), report satisfaction with healthcare (OR = 1.709), and know where to get HIV testing (OR = 1.384), but also showed higher odds of accepting unprotected sex for incentives (OR = 1.616). These distinctions are not merely behavioral but have direct implications for HIV risk mitigation strategies. While non-injection users show more proactive health-seeking behaviors, their greater likelihood of engaging in unprotected sex for incentives highlights a different axis of HIV vulnerability. Previous studies suggest that sex workers who inject drugs face elevated health risks, including higher rates of HIV and injection-related infections, due to overlapping structural and behavioral vulnerabilities [41], [43]. PWIDs and SWs are disproportionately affected by blood-borne viruses (BBVs), including HIV and hepatitis C [37–38]. They often face substantial barriers to accessing health and social care services, including but not limited to stigma, criminalization, and lack of culturally appropriate support, further complicating their health vulnerabilities [46]. Additionally, both groups are frequently underrepresented in surveillance systems and research data, leading to gaps in evidence that hinder the development of effective, tailored interventions [47]. These findings align with the syndemic theory, which suggests that substance use, HIV risk, and structural violence co-occur and mutually reinforce one another within marginalized populations [4].

Entry into sex work is associated with a significant increase in substance use, particularly as a coping mechanism for trauma and stress [48]. Furthermore, our qualitative data emphasized substance use as a coping response to stigma, trauma, and marginalization. Our findings show that these emotional burdens are not isolated; rather, they directly correlate with reduced health service access and lower exposure to HIV prevention tools, particularly among injection users. Participants described emotional distress, regret, and reliance on substances by peers to manage internalized shame, in line with existing literature reporting that street-based sex workers frequently face complex health and social challenges, driven by high rates of heroin, cocaine, and injection drug use, poor treatment outcomes, elevated morbidity and mortality, mental and physical health issues, as well as exposure to sexual and physical violence and homelessness [40]. Additionally, sex workers may use stimulants to suppress appetite, reduce the need for rest, and stay alert during extended work hours, which might limit their ability to access or prioritize healthcare services [49].

Substance use was also associated with structural exclusion. Increased alcohol and tobacco use correlated with greater experiences of healthcare discrimination (alcohol: Cramér’s V = 0.42) and immigration-related barriers (tobacco: V = 0.34). Increased substance uses also corresponded with higher rates of annual HIV testing, but less frequent general healthcare utilization, suggesting that testing may be episodic or institutionally triggered rather than part of regular preventive care. These structural exclusions were statistically significant in our sample, particularly for alcohol and tobacco users, who reported higher levels of stigma and immigration-related barriers to care (Cramér’s V up to 0.42). These patterns reaffirm the intersectional and multi-level marginalization experienced by individuals at the intersection of race, migration, and drug use. Although chemsex was not widely reported by participants, providers identified it as an emerging concern, with gaps in outreach for those engaging in stimulant-driven sex. It is important to consider that the emergence of chemsex phenomena signals evolving substance use trends that may be under-detected due to service blind spots. Targeted surveillance and culturally competent outreach are urgently needed to capture this hidden dimension. Overall, these results highlight the need for structural interventions—harm reduction, decriminalization, and inclusive healthcare, to address the compounded vulnerabilities of African refugee sex workers in Italy and Europe. Policymakers should prioritize low-threshold harm reduction services that are migrant-inclusive, support peer-based interventions, and decouple healthcare access from immigration enforcement. In addition, scaling culturally tailored HIV testing and chemsex harm-reduction programs is critical, although future research should address, evaluate, and pilot interventions for both groups (injection and non-injection drug users and sex workers) [48].

Our findings align with syndemic theory by demonstrating how substance use, stigma, healthcare exclusion, and immigration-related stressors interact synergistically to produce heightened HIV vulnerability. These co-occurring conditions are not independent risk factors but intersecting axes of compounded marginalization. The differential trajectories and impacts of injectable vs. non-injectable substance use further highlight syndemic complexity, where the type of use intersects with behavioral risk, service engagement, and mental health outcomes. This syndemic lens reinforces the inadequacy of siloed or behavior-only interventions and supports calls for integrated harm reduction, legal reform, and migrant-inclusive HIV programming.

This study provides robust evidence that substance use increases following entry into sex work and intertwines with structural factors to shape HIV vulnerability. The differentiation between injection and non-injection use offers nuanced insights that can inform harm reduction and healthcare delivery models. However, the study has limitations. First, causality cannot be established due to the cross-sectional nature of the survey data. Second, self-reported substance use and sexual behaviors may be affected by recall and social desirability bias. Third, while we employed mixed methods, the qualitative sample was not stratified by drug use type, limiting comparative depth. Future research should incorporate longitudinal designs, disaggregated analysis by gender identity and drug type, and explore chemsex trends among refugee sex workers more systematically. Efforts should also be made to improve the inclusion of African refugee sex workers in Italian and European surveillance systems to generate actionable epidemiological data.

### Limitations

This study has several limitations that should be considered when interpreting the findings. First, its cross-sectional design precludes causal inference between substance use trajectories and HIV vulnerability. Second, substance use and sexual behavior measures relied on self-report, which may be affected by recall and social desirability biases, particularly given the stigmatized nature of both sex work and drug use. Third, while a mixed-methods design enriched interpretation, the qualitative sample was not stratified by substance use type or gender identity, limiting comparisons across subgroups. Fourth, the classification rule labeling substances as “injectable” if they can be injected may have slightly overestimated injection prevalence; however, sensitivity analyses using alternative classifications did not materially change the results. Fifth, although recruitment through community-based organizations enhanced trust and participation, it may have introduced selection bias by underrepresenting less connected or hidden subgroups. Lastly, the lack of longitudinal follow-up restricts understanding of changes in substance use and health outcomes over time. Future studies should employ longitudinal and mixed-method designs with stratified recruitment and biomarker verification to strengthen causal interpretation and reduce self-report bias.

## Conclusion

Despite these limitations, this mixed-methods study provides novel insights into how substance use intersects with structural and behavioral HIV vulnerabilities among African refugee sex workers in Italy. This study offers compelling evidence that substance use, HIV vulnerability, and structural exclusion operate as interlinked epidemics among African refugee sex workers in Italy. Grounded in syndemic theory, our findings emphasize the mutually reinforcing nature of behavioral risks and social determinants such as stigma, legal precarity, and institutional discrimination. Differentiating between injection and non-injection drug use revealed nuanced patterns of health behavior and service engagement, underscoring the need for tailored intervention strategies. To disrupt this syndemic, policymakers and practitioners must prioritize decriminalization, culturally competent and migrant-inclusive healthcare, and low-threshold harm reduction approaches. Importantly, this study is among the first to apply a mixed-methods syndemic framework to the experiences of African refugee sex workers in a European context, a population that remains severely underrepresented in public health research. By integrating narrative and statistical insights, the study demonstrates that substance use and related HIV risks are not solely products of individual behavior but are embedded within broader systems of exclusion. These findings advance understanding of how structural violence compounds health disparities and point toward equitable, community-informed HIV prevention strategies.

## Figures and Tables

**Table 1.** Participants demographics

Demographic	n (%) or Mean ± SD
**Age (years)**	30.6 ± 5.9
**Education Level**	Secondary or more, 113 (75), Primary or less, 37 (25)
**Gender Identity**	Man, 145 (96.7) Transgender, 3 (2.0) Non-binary, 2 (1.3)
**Marital Status**	Single, 86 (57.3) Married, 53 (35.3) Divorced, 10 (6.7) Widowed, 1 (0.7)
**Number of Children**	None, 80 (53.3) One or more, 70 (46.7)
**Religious Affiliation**	Christianity, 71 (47.3) Islam, 37 (24.7) African Traditional Religion, 16 (10.7) No religion, 25 (16.7) Other 1 (0.7)
**Immigration Status**	Refugee, 131 (87.3) Others, 19 (12.7)
**Country of Origin**	Nigeria, 86 (57.3) Ghana, 16 (10.7) Cameroon, 6 (4.0) Others, 44 (29.3)
**Sexual Orientation**	Gay, 106 (70.7) Bisexual, 44 (29.3)
**Sex role**	Top, 63 (42) Bottom, 28 (18.7) Versatile, 59 (39.3)
**Employment Beyond Sex Work**	No, 118 (78.7) Yes, 38 (21.3)
**Length of Stay in Italy**	One year or more, 87 (58) Less than a year, 63 (42)

*Note.* Values are presented as n (%) unless otherwise indicated.

**Table 2.** The results of repeated ANOVA

Effect	*F(1, 149)*	*p*-value
**Time (Before vs. After)**	59.99	< 0.001
**Substance Type (Injectable vs. Non-injectable)**	397.01	< 0.001
Time * Substance Type	12.11	< 0.001

**Table 3.** Paired t-test outputs

Substance Type	Time Point	Mean (SD)	*t*	*p*-value	95% CI (Diff in Means)
**Injectable**	Before	3.01 (3.10)			
	After	5.60 (2.04)	7.82	< 0.001	[1.93, 3.24]
**Non-injectable**	Before	4.23 (3.74)			
	After	7.31 (2.36)	7.51	< 0.001	[2.27, 3.90]

**Table 4.** The results of logistic regression and multinomial logistic regression analyses

Predictor	*OR*	*p_value*	Dependent Variable
**Non-injection use**	1.482	0.015	Distance to healthcare facility
**Non-injection use**	1.384	0.036	Knowledge of where to test for HIV
**Injection use**	0.575	0.006	Notify clients if diagnosed with STI
**Non-injection use**	1.554	0.013	Notify clients if diagnosed with STI
**Injection use**	0.483	0.005	Enjoyment of sex work
**Non-injection use**	1.582	0.044	Enjoyment of sex work
**Non-injection use**	1.709	0.034	Satisfaction with healthcare quality
**Non-injection use**	1.559	0.026	STI diagnosis (ever told by provider)
**Injection use**	0.499	0.008	Unmet healthcare needs
**Non-injection use**	1.616	0.005	Engaged in incentivized unprotected sex
**Injection use**	0.646	0.024	Engaged in incentivized unprotected sex

**Table 5.** Associations Between Increased Substance Use and HIV Testing Frequency

Substance Use	*χ* ^2^	p-value	Cramér’s V	Key Pattern
**Tobacco**	20.39	< 0.001	0.37	↑ Annual testing among tobacco users
**Illicit drugs**	17.37	< 0.001	0.34	↑ Annual testing among drug users
**Alcohol**	14.35	0.002	0.31	↑ Annual testing among drinkers

**Table 6.** Associations Between Increased Substance Use and Healthcare Utilization

Substance Use	*χ* ^2^	p-value	Cramér’s V	Key Pattern
**Alcohol**	9.19	0.002	0.25	↓ Frequent healthcare access
**Illicit drugs**	6.61	0.010	0.21	↓ Frequent healthcare access

**Table 7.** Associations Between Increased Substance Use and Stigma in Healthcare Settings

Substance Use	*χ* ^2^	p-value	Cramér’s V	Key Pattern
**Tobacco**	15.65	< 0.001	0.32	↑ Stigma reports among tobacco users
**Alcohol**	25.89	< 0.001	0.42	↑ Stigma reports among drinkers
**Illicit drugs**	9.33	0.002	0.25	↑ Stigma reports among drug users

**Table 8.** Associations Between Increased Substance Use and Immigration-Related Healthcare Barriers

Substance Use	*χ* ^2^	p-value	Cramér’s V	Key Pattern
**Tobacco**	17.82	< 0.001	0.34	↑ Barriers reported among tobacco users
**Alcohol**	15.11	< 0.001	0.32	↑ Barriers reported among drinkers
**Illicit drugs**	9.28	0.002	0.25	↑ Barriers reported among drug users

**Table 9.** Integrated Findings on Substance Use, Healthcare, and HIV Vulnerability

Quantitative Finding	Qualitative Finding (from Results section)	Integrated Interpretation
Increased alcohol and illicit drug use after entering sex work were associated with reduced healthcare access (*χ*^2^ = 9.19 & 6.61, *p* < 0.01).	Participants described how substance use sometimes led to neglect of health: *“Some under the effect of substances… don’t give a damn, so for them it’s lîfe, only one, they live as they want.”*	Convergence: Both strands show substance use contributes to reduced healthcare engagement.
Substance use escalation was associated with more reports of stigma and immigration-related barriers in healthcare (*χ*^2^ range = 9.28–25.89, all *p* < 0.01).	Respondents highlighted systemic barriers and lack of targeted services: *“There are difficulties in intercepting… the population of sex workers, or people who actually use substances… [who] do not access because there is perhaps no service ready to receive them.”*	Complementarity: Quantitative associations are explained by qualitative accounts of unmet service readiness and exclusion.
Increased substance use linked to HIV testing outcomes (annual vs. voluntary testing).	Some participants only learned their HIV status in institutional contexts: *“When they enter the prison… they find out randomly they already have HIV but didn’t know.”*	Divergence: Quantitative data suggest increased testing, while qualitative findings reveal this may reflect compulsory, not voluntary, testing.

**Table 10.** Integrated Findings on Sexual Risk Behaviors and Disclosure

Quantitative Finding	Qualitative Finding (from Results section)	Integrated Interpretation
Injection drug use was associated with reduced enjoyment of sex work and disengagement from HIV prevention tools (ORs < 1, all *p* < 0.05).	Participants described negative experiences with sex under the influence: *“I sometimes get bad when I have sex under the influence of alcohol, and when I wake up, and see the person is not up to standard. I feel bad about it, very, really bad.”*	Convergence: Both strands highlight how substance use reduces satisfaction and agency in sex work.
Non-injection users had higher odds of disclosure and engagement with services (ORs > 1, *p* < 0.05).	Peers were described as turning to substances due to stigma: *“I meet with a Nigerian guy who got into drugs because of stigma from his friends… he said no, that’s the only thing that helps him.”*	Complementarity: Quantitative differences may reflect varying coping strategies, where noninjection users stay more connected to social networks and services.
Non-injection users had higher odds of engaging in incentivized condomless sex (OR = 1.616, *p* = 0.005).	Some participants noted pressures from clients under the influence: *“dangerous encounters with clients under the influence of drugs.”*	Convergence: Both strands underscore economic and situational pressures driving sexual risk, especially linked to substance use contexts.
